# Fasting plasma glucose concentrations for specified HbA1c goals in Korean populations: data from the Fifth Korea National Health and Nutrition Examination Survey (KNHANES V-2, 2011)

**DOI:** 10.1186/s13098-016-0179-8

**Published:** 2016-08-30

**Authors:** Sangmo Hong, Jun Goo Kang, Chul Sik Kim, Seong Jin Lee, Chang Beom Lee, Sung-Hee Ihm

**Affiliations:** 1 Division of Endocrinology, Department of Internal Medicine, Hallym University Dongtan Sacred Heart Hospital, 7, Keunjaebong-gil,Hwaseong-si, Gyeonggi-do, 445-907, Republic of Korea; 2 Division of Endocrinology and Metabolism, Department of Internal Medicine, Hallym University Sacred Heart Hospital, Hallym University College of Medicine, 896 Pyeongchon-dong, Dongan-gu, Anyang, Gyeonggi-do Republic of Korea; 3 Department of Endocrinology and Metabolism, Hanyang University Guri Hospital, Gyomun 1(il)-dong, Guri-si, Gyeonggi-do 471-701 Republic of Korea

**Keywords:** Diabetes mellitus, Diagnosis, Glucose, Hemoglobin A, Glycated

## Abstract

**Aims:**

To examine the correlation between the fasting plasma glucose and HbA1c levels using regression equation and to assess the average fasting plasma glucose levels for the specific HbA1c (A1C) goals in the patients with diabetes using each A1C-and fasting plasma glucose-based diagnostic criteria.

**Methods:**

This study included data from 4481 participants with A1C and fasting plasma glucose, but with no diabetic medications in the Korean National Health and Nutritional Examination Survey 2011. The correlation between fasting plasma glucose and A1C was examined using linear regression models.

**Results:**

The A1C levels corresponding to the fasting plasma glucose of 5.5 and 7 mmol/L were 5.75 and 6.42 %. However, in the subjects with diabetes diagnosed by the A1C criteria only, 5.5 and 7 mmol/L in the fasting plasma glucose predicted A1C of 6.49 and 7.14 % respectively. The average fasting plasma glucose levels to achieve specified A1C levels of 5.0–5.9, 6.0–6.9, 7.0–7.9, 8.0–8.9, and 9.0–9.9 % were 5.1, 6.1, 7.7, 8.8 and 11.2 mmol/L, respectively.

**Conclusions:**

The association between A1C and fasting plasma glucose levels is in concordance with the existing criteria for diagnosis of diabetes. However, the average fasting plasma glucose concentrations to achieve targeted A1C may be lower than those in western populations.

## Background

The incidence of type 2 diabetes is becoming an emergency worldwide, particularly in East Asia, potentially reaching a pandemic level in the very near future [1]. Although a significant progress has been made in reducing cardiovascular risk factors among Korean patients with diabetes [2], type 2 diabetes creates a considerable amount of economic and social burdens in Korea due to its long-term and wide-ranging multiple chronic complications [3, 4]. Therefore, it is important to prevent these complications through its early diagnosis and prevention.

Fasting plasma glucose (FPG) should be measured for the purpose of screening or diagnosis for impaired fasting glucose and diabetes (hyperglycemic states) [5]. However, there are certain limitations with it: patients must fast more than 8 h before getting screened for diabetes and there are numerous factors (e.g., stress, acute illness) that can alter glucose concentrations [6]. Hemoglobin A1c (A1C) is a standard monitoring test for long-term glycemic control and was recently recommended for the diagnosis of diabetes as well although the generalization of the A1C diagnostic level compared with a glucose-based diagnosis may still be debated across ethnic groups [7]. In 2010, The American Diabetes Association (ADA) has recommended the A1C as a diagnostic tool for diabetes [8, 9] and in 2011, Korean Diabetes Association (kDa) has recommend the A1C as a diagnostic criterion for diabetes in clinical practice guidelines for type 2 diabetes [10, 11]. Recent study and recommendation showed average fasting glucose concentrations associated with specific A1C levels [12, 13]. This result can help the clinicians explain the relationship between fasting glucose concentrations and specific A1C levels more easily to the patients, but no study on this relationship has been made in Korea. Although there have been several hospital-based studies that have evaluated appropriate FPG and A1C cutoff levels for identifying patients with hyperglycemic states [14–16], it is thought that larger epidemiological studies are required to confirm the correlation between A1C and FPG for the diagnosis of diabetes in Korean population. We conducted this study to examine the relationship between the FPG and A1C for diabetes diagnosis (using the regression equation) and to access the average FPG concentrations associated with specific A1C levels in diabetic patients (using each A1c-based and FPG-based diagnostic criteria) [4, 11, 17, 18] in the recent national representative sample of the Korean adult population.

## Methods

The Korea National Health and Nutrition Examination Survey (KNHANES), a nationwide cross-sectional survey and representative of non-institutionalized civilians in Korea, is conducted periodically by the Korea Centers for Disease Control and Prevention (KCDC). KNHANES was initiated in 1998 and was designed to provide comprehensive information about Koreans’ health status, health behavior, and nutritional status [19]. Data from the second year (2011) of KNHANES V (including A1C and FPG), was used in this cross-sectional analysis. To produce an unbiased national estimate, a sample weight was assigned for the participating individuals to represent the Korean population. Sampling weights were designated by the Korean Centers for Disease Control and Prevention and were constructed to account for the complex survey design, survey non-response, and poststratification [19].

From an initial total of 8518 men and women, 6066 (2677 men and 3389 women) with A1C and FPG, but without previous pharmacological therapy, were evaluated. From these 6066 persons, 1585 were excluded for the following reasons: 82 lacked underlying disease information; 707 were below 18 years old; 262 had fasting time less than 10 h; 366 had anemia defined as a hemoglobin value <130 g/L in men (n = 67) and <120 g/L in women (n = 299); 8 were pregnant; 14 had the estimated glomerular filtration ratio (eGFR) defined by the modification of diet in renal disease (MDRD) study equation less than 60 mL/min/1.73 m^2^; 9 had liver cirrhosis; and 137 had cancer of different kinds. Therefore, the final number of participants used in this analysis was 4481 and the total number was estimated to be 29,186,176 after a sample weight was assigned for the participating individuals to represent the Korean population. All examination (or testing) protocols were approved by the Institutional Review Board of Korea Centers for Disease Control and Prevention with each subject providing written informed consent before participating and the study protocol using this date was reviewed and approved by the institutional review board of Hallym University Sacred Heart Hospital.

Blood samples of all subjects were collected and the specimens were immediately centrifuged, aliquoted, frozen at −70 °C, and moved to the central laboratory (Neo-DIN Medical Institute, Seoul, South Korea), where they were analyzed within 24 h. The FPG concentrations were measured using an automated analyzer with an enzymatic assay (Pureauto S GLU: Daiichi, Tokyo, Japan) and the A1C was measured using high performance liquid chromatography (HLC-723G7: Tosoh, Yamaguchi, Japan). The thresholds for the diagnosis of diabetes were 7.0 mmol/L or greater by the FPG criteria, and 6.5 % or higher by the A1C criteria.

The statistical analysis was performed using SPSS (Chicago, IL) and weighted to the Korean population to provide nationally representative estimates. The baseline characteristics are presented as the means for continuous variables, and proportions for categorical variables. The association between fasting blood glucose and A1C was examined using linear regression models.

## Results

**Fig. 1:**
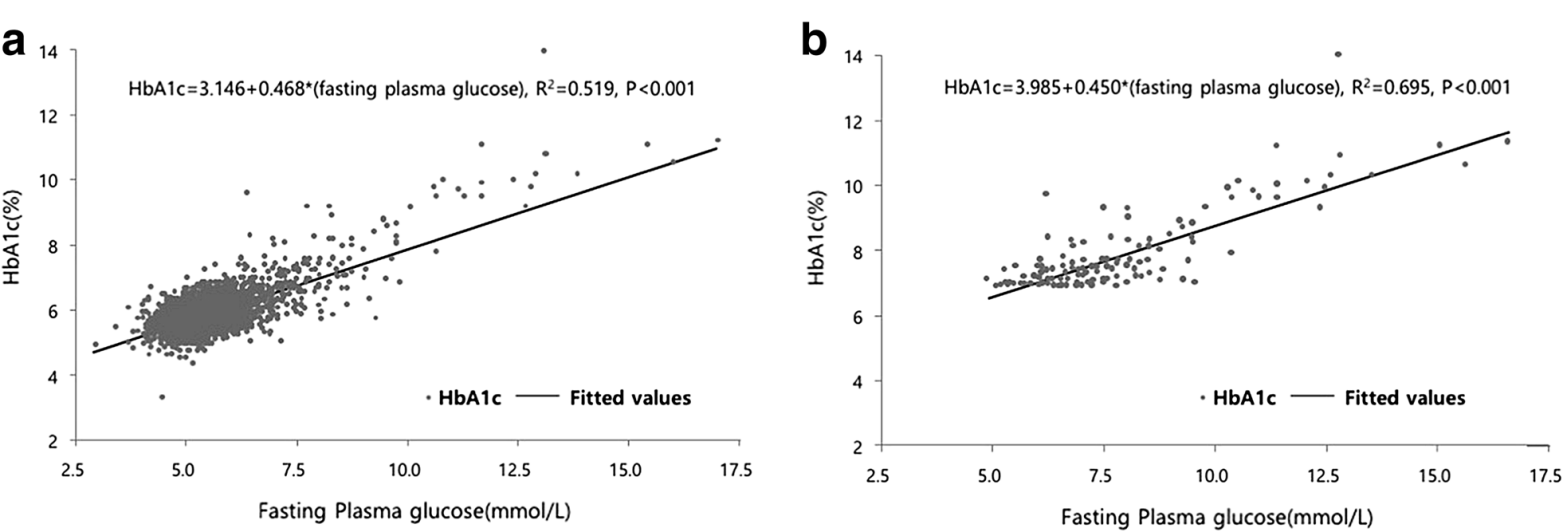
Linear regression analysis of fasting plasma glucose and HbA1c in this study population (**a**) (n = 4481) and in persons with diabetes by HbA1c criteria (**b**) (n = 199)

The characteristics of the study population are presented in Table 1. The study subjects included 1992 men (estimated to be 15,168,273) and 2489 women (estimated to be 14,017,903). The average FPG value was 5.2 ± 0.8 mmol/L (ranging from 2.8 to 16.2 mmol/L) and the average A1C value was 5.6 ± 0.5 % (ranging from 3.1 to 13.6 %). Using data from KNHANES, the results of the simple linear regression analysis between the A1C level and FPG are summarized in Fig. 1. The prediction model between the A1C and FPG in this study population, expressed as A1C = 3.146 + 0.468 × FPG (R^2^ = 0.519, p < 0.001), is shown in Fig. 1a. The A1C level increased by approximately 0.47 % per the increment of 1.0 mmol/L in FPG. In the cases with diabetes diagnosed only by the FPG criteria,, the Pearson correlation coefficient (*r*) was 0.726 and the prediction model was expressed as A1C = 2.033 + 0.612 × FPG (R^2^ = 0.686, p < 0.001) while the change in A1C per the increment of 1.0 mmol/L in FPG was 0.61 %. The correlation between the A1C level and the FPG in participants with diabetes diagnosed only by the A1C criteria, shown in Fig. 1b, is expressed as is A1C = 3.985 + 0.45 × FPG, (R^2^ = 0.695, p < 0.001). The change in A1C per the increment of 1.0 mmol/L in FPG was 0.45 % in these participants.

**Table 1:** Clinical characteristics of the study population (n = 4481)

	Mean ± standard deviation	Minimum	Maximum
Age (years)	48.6 ± 16.3	18	90
Sex (women %)	55.5		
Waist circumflex (cm)	81.2 ± 10.1	52	137
Body mass index (kg/m^2^)	23.6 ± 3.4	13	45
Systolic blood pressure (mmHg)	118 ± 17	79	205
Diastolic blood pressure (mmHg)	76 ± 10	40	119
FPG (mmol/L)	5.2 ± 0.8	2.8	16.2
A1C (%)	5.6 ± 0.5	3.1	13.6
Total cholesterol (mmol/L)	5.0 ± 0.9	2.4	14.3
Triglyceride (mmol/L)	3.4 ± 2.7	0.2	21.9
LDL cholesterol (mmol/L)	2.9 ± 0.8	0.6	8.1

*A1C* hemoglobin A1c, *FPG* fasting plasma glucose, *LDL* low density lipoprotein

Based on these calculations, the correspondence of A1C to specific FPG were shown in Table 2. We found similar results of the linear regression between all study population and diabetics diagnosed only by the FPG criteria. However, A1C levels in the subjects diagnosed only by FPG criteria are slightly lower than those found in all study population. A FPG of 5.5 mmol/L predicted an A1C of 5.75 % in the study population and of 5.43 % in the subjects diagnosed only by FBS criteria. A FPG of 7 mmol/L also predicted an A1C of 6.42 and 6.32 % respectively. However, in the subjects with diabetes diagnosed only by the A1C criteria, 5.5 and 7 mmol/L predicted 6.49 and 7.14 % in the A1C respectively. From these regression equations, FPG levels for A1C of 6.5 % were calculated at approximately 7.3 mmol/L in diabetics diagnosed by the FPG criteria, and 5.5 mmol/L only by A1C criteria. Mean fasting plasma glucose concentrations for specific A1C groups are summarized in Table 3. The average FPG needed to achieve specified A1C levels of lower than 5, 5.0–5.9, 6.0–6.9, 7.0–7.9, 8.0–8.9, and 9.0–9.9 % and more than 10 % were 4.8 mmol/L with 95 % CI 2.8–6.8, 5.1 mmol/L (3.3–8.9), 6.1 mmol/L (4.0–8.7), 7.7 mmol/L (5.4–10.2), 8.8 mmol/L (7.4–12.1) 11.2 mmol/L (6.1–13.2) and 13.9 mmol/L (11.1–16.2) respectively. These results showed that mean FPG concentrations for specified A1C levels in this study were lower than those in previous western studies [12, 13] (Table 3).

**Table 2:** A1C estimated from the regression analysis

FPG (mmol/L)	Regression estimated A1C (%)		
	Study population	Diabetes by FPG criteria only	Diabetes by A1C criteria only
5.3	5.65	5.30	6.39
5.5	5.75	5.43	6.49
5.6	5.77	5.47	6.51
6.3	6.08	5.88	6.81
6.7	6.26	6.11	6.99
7	6.42	6.32	7.14
7.2	6.50	6.42	7.21
7.3	6.55	6.49	7.26

*A1C* hemoglobin A1c, *FPG* fasting plasma glucose

**Table 3:** Mean fasting plasma glucose levels (95 % CI) for specified A1C levels in the ADAG study and KNHANES 2011

A1C (%)	KNHANES 2011^a^		ADAG study [12]^b^
	Mean FPG		Mean fasting glucose
	Mean (mmol/L) ± SD	Range	
<5	4.8 ± 0.4	2.8–6.8	
5	5.1 ± 0.5	3.3–8.9	
6	6.1 ± 0.9	4.0–8.7	
6.0–6.4	5.9 ± 0.8		6.8^a^
6.5–6.9	6.8 ± 1.1		7.9
7	7.7 ± 1.1	5.4–10.2	
7.0–7.4	7.5 ± 1.0		8.4
7.5–7.9	8.0 ± 1.1		9.3
8	8.8 ± 1.2	7.4–12.1	9.9^c^
9	11.2 ± 1.3	6.1–13.2	
≥10	13.9 ± 1.8	11.1–16.2	

Data are expressed as mean (±SD)

*ADAG* A1c-derived average glucose, *A1C* hemoglobin A1c, *FPG* fasting plasma glucose, KNHANES Korea National Health and Nutrition Examination Survey

^a^The range of specified A1C for this fasting glucose level (6.8 mmol/L) is from 5.5 to 6.49 %

^b^Modified from Wei et al. Diabetes Care 2014;37:1048–1051 [12]. These estimates are based on the SMBG data from 470 of the ADAG study participants (237 with type 1 diabetes and 147 with type 2 diabetes) to determine the average fasting blood glucose for specified HbA1c groups

^c^The range of specified A1C for this fasting glucose level (9.9 mmol/L) is from 8.0 to 8.5 %

## Discussion

The results of this study supported a close association between A1C and FPG levels for the diagnosis of diabetes for the Korean population, which is in concordance with the recent recommendations [4, 11, 17, 18]. The A1C levels corresponding to a FPG of 5.5 mmol/L were 5.75 % in all study population and 5.43 % in participants with diabetes diagnosed by FPG-based diagnostic criteria only. The A1C levels corresponding to a FPG of 7 mmol/L were also 6.42 and 6.32 % respectively. This suggests that the established FPG criteria for the diagnosis of prediabetes and diabetes is relatively valid in Korean population. However, it is thought that there are several issues in order to adopt these diagnostic criteria for diabetes in Korea.

First, the relationship of A1C and blood glucose with the risk of diabetic retinopathy (DR) should be supported by epidemiological studies in Korean population. The association between chronic glucose concentrations and microvascular complications, particularly DR, has been the basis for the diagnosis of diabetes. The diagnostic A1C cutoff in diabetes (≥6.5 %) is associated with an optimal point for the prevalence of DR, as are the diagnostic values for FPG [8]. However, the KDA has adopted the diagnostic criteria for type 2 diabetes from the ADA recommendation so far because there has been no prospective epidemiological study in Korean population to demonstrate an association between blood glucose level and the development of DR [11]. A recent study provided important evidence that A1C threshold of 6.5 % is a suitable test for the diagnosis of diabetes mellitus in three major Asian ethnic groups, but this study also showed that specific A1C levels had different sensitivity and specificity in these populations [20]. Therefore, A1C should be used thoughtfully and in combination with traditional glucose criteria for screening and diagnosis of diabetes, although the reasons for these differences remain unknown [21]. One Korean community-based cross-sectional study showed that the A1C cutoff of 6.5 % allowed the proper detection of DM and supported the appropriate use of A1C for the diagnosis of diabetes [22]. However, the recent nationwide epidemiological study from the 2011 KNHANES demonstrated that both FPG and A1C levels were closely associated with DR [23]. This study showed that the optimal glycemic and A1C levels for detecting DR were 6.3 mmol/L for FPG and 6.2 % for A1C in Korean population suggesting that current diabetes diagnostic cutoffsshould be lower for Koreans [23]. The results of our study also show that a FPG of 6.3 mmol/L predicted an A1C of approximately 6.1 %, supporting the previous study in Korean population.

Second, the patients’ race/ethnicity may influence A1C [6, 24, 25]. A recent study showed that African Americans have higher A1C levels than non-Hispanic whites after adjustment for fasting glucose levels, but had lower levels of 1,5-anhydroglucitol suggesting that their postprandial glucose levels may be higher [26]. However, there was no report for correlation between FPG and specific A1C levels in Asian population after 2010 ADA indication that A1C should be performed according to the method certified by the National Glycohemo-globin Standardization Program (NGSP) and standardized to the diabetes control and complication trial (DCCT) assay [8, 9]. In the present study, the A1C levels corresponding to a FPG of 5.5 and 7 mmol/L were 6.49 and 7.14 % respectively in Korean population with newly diagnosed diabetes using A1C-based diagnostic criteria only. These findings are inconsistent with those in participants who are diagnosed by FPG-based diagnostic criteria only in this study and are also inconsistent with those in recent recommendations [4, 11, 17, 18]. In fact, it is difficult to directly compare mean FPG levels for specified A1C levels in the A1c-derived average glucose (ADAG) study and our study because the ADAG study was a multicenter observational study of serial FPG for specified A1C levels. However, it is reported that participants with an elevated A1C but “nondiabetic” FPG may have the likelihood of showing higher postprandial glucose levels or elevated glycation rates [4] and the results of our study rather supported these hypothesis in Korean population. Therefore, we need to pay more attention that the diabetic patients who are diagnosed by A1C-based criteria only have lower FPG compared to those who are diagnosed by FPG-based criteria only. It also needs to be noted that the patients diagnosed by A1C-based criteria only can have higher postprandial glucose levels than those who are diagnosed by FPG-based criteria only.

Third, it is important that FPG is accurately measured for the screening or diagnosis of prediabetes and diabetes, although it was reported that it would be inappropriate to use the fasting glucose criteria alone for screening diabetes and the 2-h glucose seems to be a particularly important diagnostic tool in Asian populations [27]. In fact, most Korean people take regular (biennial) medical examinations at the National Health Insurance Corp including fasting glucose measurement [28]. Any suspicious findings on the results can be sufficient to suggest that they need a doctor’s exam or inspections for an accurate diagnosis and any necessary treatments. However, many Korean people think that prediabetes and diabetes are screened as a result of these examinations even though health screening is not for diagnosis, and they are not worried about these hyperglycemic states if normal or slightly elevated serum glucose levels are measured. Furthermore, many laboratories including the National Health Examination measure serum glucose even though guidelines rather recommend measurement of plasma because large-scale medical checkups require a simple and cost-effective sample collection method [6]. Our previous hospital-based report using data from 2028 persons for the evaluation of metabolic abnormalities including impaired fasting glucose or dyslipidemia suspected through health examinations in the National Health Insurance Corp. or by personal medical checkup showed that 22.1 % had newly diagnosed diabetes according to FPG. Even, among participants with normoglycemia according to serum glucose, 14.2 % had newly diagnosed diabetes according to FPG diagnostic criteria [29]. It is thought that these findings may be one of the important reasons why the diagnosis of diabetes, which is the fifth-leading cause of death in Korea, could be underestimated.

Our study had several limitations. First, this study was based on a cross-sectional analysis. Second, participants self-reported whether they were taking any glucose-lowering medications and from this, recall bias may exist. Third, because we did not have the results of 75 g oral glucose tolerance test (OGTT), the diagnosis of diabetes among our participants may be underestimated and an association between A1C and 2 h postprandial glucose levels in Korean population with newly diagnosed diabetes by only A1C-based diagnostic criteria was not assessed. Fourth, we excluded participants with anemia but not hemoglobinopathies. This, however, does not seem to be an issue as the prevalence of hemoglobinopathy is very low in Korea [30, 31]. Finally, some clinical conditions that could influence the interpretation of A1C (such as inflammation or change of diet) were not included in this study. Despite these limitations, present study, to our knowledge, is the first epidemiological report showing the correlation between FPG concentrations and A1C levels after using A1C for the diagnosis of diabetes, and the association between the mean FPG concentrations and specific A1C levels. However, this study also shows a great difference in the correlation between FPG and A1C values in the two groups diagnosed only by FPG and only by A1C criteria respectively. These results require further study to compare the characteristics of individuals who are newly diagnosed by either only A1C- or FPG-based criteria. Large-scale longitudinal study should be made to investigate the association between levels of A1C and FPG with incident diabetic retinopathy and to determine the optimal values of A1C and FPG for detecting incident diabetic retinopathy in the Korean population.

## Conclusions

Our study showed that a close association between A1C and FPG levels is in concordance with the previous criteria for diagnosis of diabetes. However, the average FPG concentrations to achieve targeted A1C in the subjects with diabetes diagnosed only by A1C-based criteria may be lower than those in western populations. Therefore, we need to pay attention that patients diagnosed by A1C-based criteria only can have higher postprandial glucose levels than those who are diagnosed by FPG-based criteria only, in real-life practice.

## Abbreviations

ADA: American Diabetes Association
ADAG: A1c-derived average glucose
DCCT: diabetes control and complication trial
DR: diabetic retinopathy
eGFR: estimated glomerular filtration ratio
FPG: fasting plasma glucose
A1C: hemoglobin A1c
KCDC: Korean Centers for Disease Control and Prevention
kDa: Korean Diabetes Association
KNHANES: Korea National Health and Nutrition Examination Survey
MDRD: modification of diet in renal disease
NGSP: National Glycohemoglobin Standardization Program
OGTT: oral glucose tolerance test
